# Metagenomics and metabarcoding experimental choices and their impact on microbial community characterization in freshwater recirculating aquaculture systems

**DOI:** 10.1186/s40793-023-00459-z

**Published:** 2023-02-14

**Authors:** Jessica Rieder, Adamandia Kapopoulou, Claudia Bank, Irene Adrian-Kalchhauser

**Affiliations:** 1 Department of Infectious Diseases and Pathobiology, Vetsuisse Faculty, Institute for Fish and Wildlife Health, University of Bern, Länggasstrasse 122, 3001 Bern, Switzerland; 2 Division of Theoretical Ecology and Evolution, Institute of Ecology and Evolution, University of Bern, Baltzerstrasse 6, 3012 Bern, Switzerland; 3 Swiss Institute of Bioinformatics, Quartier Sorge - Batiment Amphipole, 1015 Lausanne, Switzerland

**Keywords:** 16S rRNA gene, Amplicon sequencing, Shotgun metagenomics, DADA2, ASVs, MiSeq, PacBio, Short-reads, Long-reads

## Abstract

**Background:**

Microbial communities in recirculating aquaculture systems (RAS) play a role in system success, nutrient cycling, and water quality. Considering the increasing socio-economic role of fish farming, e.g., regarding food security, an in-depth understanding of aquaculture microbial communities is also relevant from a management perspective, especially regarding the growth, development, and welfare of the farmed animal. However, the current data on the composition of microbial communities within RAS is patchy, which is partly attributable to diverging method choices that render comparative analyses challenging. Therefore, there is a need for accurate, standardized, and user-friendly methods to study microbial communities in aquaculture systems.

**Results:**

We compared sequencing approach performances (3 types of 16S short amplicon sequencing, PacBio long-read amplicon sequencing, and amplification-free shotgun metagenomics) in the characterization of microbial communities in two commercial RAS fish farms. Results showed that 16S primer choice and amplicon length affect some values (e.g., diversity measures, number of assigned taxa or distinguishing ASVs) but have no impact on spatio-temporal patterns between sample types, farms and time points. This implies that 16S rRNA approaches are adequate for community studies. The long-read amplicons underperformed regarding the quantitative resolution of spatio-temporal patterns but were suited to identify functional services, e.g., nitrification cycling and the detection of pathogens. Finally, shotgun metagenomics extended the picture to fungi, viruses, and bacteriophages, opening avenues for exploring inter-domain interactions. All sequencing datasets agreed on major prokaryotic players, such as *Actinobacteriota*, *Bacteroidota*, *Nitrospirota*, and *Proteobacteria*.

**Conclusion:**

The different sequencing approaches yielded overlapping and highly complementary results, with each contributing unique data not obtainable with the other approaches. We conclude that a tiered approach constitutes a strategy for obtaining the maximum amount of information on aquaculture microbial communities and can inform basic research on community evolution dynamics. For specific and/or applied questions, single-method approaches are more practical and cost-effective and could lead to better farm management practices.

**Supplementary Information:**

The online version contains supplementary material available at 10.1186/s40793-023-00459-z.

## Introduction

Recirculating aquaculture systems (RAS) are a valuable alternative to the limited sustainable capacity of capture fisheries. They are discussed as a long-term sustainable offset for capture fisheries [1] and a means to meet the nutritional demand for high-quality animal protein. RAS cultivate freshwater species such as rainbow trout (*Oncorhynchus mykiss*), pike-perch (*Stizostedion lucioperca*), Arctic char (*Salvelinus alpinus*), and sturgeon (order *Acipenseriformes*) [2] and range from small privately-owned enterprises to industrial-sized corporations. The indoor, closed-circuit design of RAS provides independence from seasonal conditions, allows for biosecurity measures, and reduces the product-to-market distance when situated inland [3].

Microbial communities in RAS play a crucial role in overall system success, nutrient cycling, water quality, and animal health [1, 4–12]. These communities are often actively maintained in the biofilter section of the system, which is designed to maximize the surface area with sand, granulated active carbon, or synthetic carrier material. Biofilter microbial communities perform various services, such as removing toxic metabolic products (e.g., ammonia, nitrite, nitrate, sulfide, and sulfate) and organic waste. Some prominent representatives of the oxidizing ammonia genera found in RAS biofilters are *Nitrosomonas*, *Nitrosospharea*, and *Nitrosospira* [13], as well as ammonia-oxidizing archaea and *Nitrotoga* species [14, 15].

Conversely, pathogenic components of microbial communities in RAS constitute a significant challenge for the fish farm industry. Fish-related disease outbreaks threaten the livelihood of farmers and food security [16] and incur an estimated $6 billion loss yearly [17] due to stock loss. Also, water-associated off-flavoring bacterial groups may adversely impact the quality of the final product [13]. Different management approaches, such as cleaning and disinfection regimes, aim to reduce opportunistic pathogen species such as *Aeromonas* or *Flavobacterium* [18] but could potentially open niches for pathogenic species and promote undifferentiated microbial growth.

Managing microbial communities in RAS is not straightforward and poses complex challenges. It has been proposed that monitoring and targeted manipulation of RAS microbial communities, based on a thorough characterization of interactions and community dynamics, may improve aquaculture management strategies [19–21]. However, RAS microbial research lags behind compared to other microbe-dependent industries, such as wastewater treatment. Furthermore, the interactions between different compartments, management operations, microbial community structure, and how community assemblages differ across facilities are only beginning to be understood [1]. Previous microbial studies have analyzed the biofilter communities in RAS farming lumpfish (*Cyclopterus lumpus* L.) [8], Atlantic salmon (*Salmo salar*), Pacific white shrimp (*Litopenaeus vannamei*), half-smooth tongue sole (*Cynoglossus semilaevis*) and turbot (*Scopthalmus maximus*) [22], but have not investigated other RAS compartments. Furthermore, inter-study comparisons are problematic because non-standardized protocols (e.g., DNA extraction, amplification, or taxonomic assignment) impact the results and conclusion [23–27]. Lastly, global studies are scarce [28], so the characterization of RAS microbial community patterns and keystone taxa remains incomplete.

In recent years, next-generation sequencing technology has led to various methods by which microbiomes can be studied. Three commonly used methods are short- and long-read sequencing, targeting the 16S gene, and shotgun metagenomics, which targets all sequences within a sample. Short-amplicon sequencing requires primers that may target one or multiple variable regions of the genes. The major drawback of short-amplicon sequencing is the lack of resolution required for species identification. Also, primer choice can introduce biases for or against certain taxonomic groups [9, 23, 29]. Long-amplicon sequencing targets all variable regions of the 16S gene, thus increasing resolution for species identification and eliminating primer choice biases. Unfortunately, both short- and long-amplicon 16S sequencing mainly target bacteria and omit other microbes, such as fungi and archaea. Shotgun metagenomics, a primer-free method, targets all sequences within a sample, allowing for the identification of all organisms present at a sufficient frequency. However, low signal-to-noise ratios may interfere with the species-level differentiation of genetically similar species. Recent studies have started combining different sequencing approaches to reduce sequencing costs, increase resolution, and gain broader knowledge than any singular method could provide [30, 31].

**Fig. 1:**
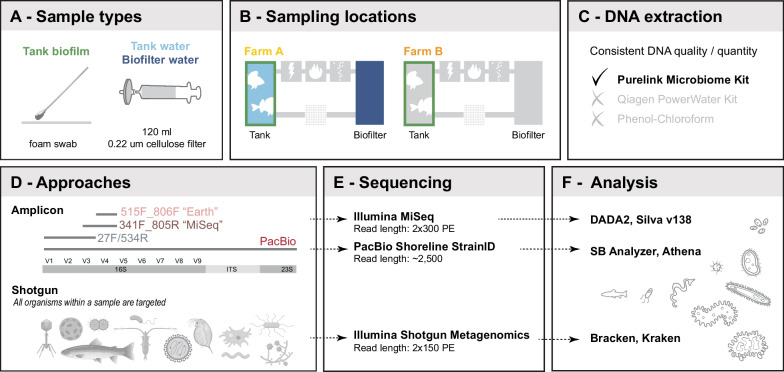
Study design and experimental steps. **A** Three types of samples were taken. Tank biofilm was collected by rubbing a foam swab against the sidewall of a tank. Tank and biofilter water was collected by filtering 120 ml of water through a 0.22 um cellulose filter. **B** Samples were taken at two farms, A and B. In farm A, all three sample types were collected. In farm B, sampling focused on tank biofilm. **C** Three DNA extraction methods were compared. The Purelink Microbiome Kit outperformed other DNA extraction methods in quality, quantity, and consistent yield. **D** Two amplicon approaches (short and long reads) and an amplification-free shotgun approach were used. **E** Short amplicons were sequenced on an Illumina MiSeq with a v3 2 × 300PE kit. Long amplicons were sequenced with the PacBio Shoreline StrainID kit, producing an average read length of 2500 bp. Shotgun sequencing was performed with a 2 × 150PE Illumina kit. **F** Illumina MiSeq sequencing data were processed with the DADA2 pipeline, and ASVs were blasted against the SILVA v138 database for taxonomic assignment. PacBio long-read data was processed with the SBAnalyzer program and taxonomically assigned using Athena. Shotgun metagenomics sequencing data were processed with an in-house pipeline that uses the Kraken-Bracken method

This study investigates the effect of sampling and analysis strategies on the inference of microbial community composition in RAS. We collected samples from two freshwater RAS to compare the ability of four primer sets, a primer-free approach, and three sequencing approaches (Fig. 1) to identify key microbial dynamics and improve future sampling and methods decisions. First, we show that primer-specific results at early analysis steps do not lead to distinct biological conclusions. Second, we demonstrate that 16S short-read sequencing is sufficient to detect spatio-temporal developments and dynamics in the context of a RAS system. Finally, we evaluate the ability of the different sequencing approaches to describe the spatio-temporal patterns and identity of microbials in different compartments of RAS, followed by a discussion of the distinct value of each sequencing approach for different research questions and farm management.

## Materials and methods

### Sampling sites

The study includes two commercial-size Swiss RAS farms (A and B) with distinct ownership and operational management procedures. Farm A breeds perch (*Perca fluviatilis*), raising offspring from egg to approximately 15 g and features several life-stage-specific circuits with independent filtration systems. Fish are moved to the next circuit when they reach a certain cutoff weight. Fish of approximately 10–15 g are raised in 13.2 m^3^ tanks at a stocking density of around 30 kg/m^3^. After each batch, a stringent disinfection regimen is applied. First, the biofilter is disconnected from the circuit to protect the microbial community from disinfection solutions. Next, the tanks are emptied, followed by a four-step cleaning regimen, (1) a high-power jet wash with hot water, (2) brushing down the tank walls and floor with soap, (3) a static acid–base treatment of the tanks and pipes with neutralizing steps in between, and (4) spraying the tanks with alcohol. Finally, the tanks are dried entirely before refilling and restocking the next batch of fish. Farm A uses multiple feed brands depending on the life stage of the fish (Bernaqua, BioMar, and Alltech Coppens).

Farm B is situated > 100 km from Farm A in a different catchment. Farm B raises two fish species: perch, obtained from Farm A at around 15 g, and pike-perch (*Sander lucioperca*), obtained at the fingerling stage from another provider. Both species are raised to slaughter weight within a single circuit in concrete tanks (120 m^3^). The stocking density varies between 30 and 60 kg/m^3^ based on the size of the fish. Cleaning regimens are applied once a tank is emptied. However, there is no strict cleaning disinfection timeline because of grading and moving the fish into new tanks, which might already be occupied. The disinfection protocol consists of (1) washing the empty tank with high-pressure hot water and (2) spraying Virkon S as a disinfection solution, followed by refilling with water and stocking with the next batch of fish. Farm B feeds with Alltech Coppens Supreme pellets of varying sizes according to fish size. Both farms use agitated biofilters with floating plastic biofilter carriers to supply the necessary surface area to foster microbial communities. Farm identities and locations are confidential.

### Sample types

Three sample types were collected: tank biofilm, tank water, and biofilter water (Fig. 1A). First, biofilm samples were collected with a sterile, single-use foam swab (Merck—product was discontinued) by rubbing one side of the swab back and forth approximately ten times across a ~ 10 × 10 cm area of the tank wall about 6 cm below surface water level and repeating the procedure on the same area with the other side of the swab. After swabbing, the swab was placed into a 2 ml Eppendorf tube, the stick was broken off, and the closed 2 ml tube was stored on ice. Biofilm replicates were taken with an approximately 2 cm gap between them. Next, using a sterile 500 ml plastic beaker, 500 ml of water were collected from the same tank as the tank biofilm sample, followed by on-site filtering of 120 ml of water using a 60 ml sterile, single-use syringe (Faust) and a 0.22 um mixed cellulose filter (Millipore, Merck) contained in a Whatman 47 mm plastic filter holder (Whatman, Merck). Replicates were taken from the same beaker, thoroughly mixing the water before the replicate was sampled. After filtration, the filters were placed in a 2 ml Eppendorf tube and stored on ice. Finally, biofilter water samples were collected, with a new sterile beaker, in the same way and from the same circuit as tank water and biofilm samples. All samples were transported back to the Institute for Fish and Wildlife Health, University Bern, on ice and stored at − 80 °C until further processing.

### Sampling scheme

The sampling scheme aimed to maximize insights into differences and similarities between replicates, sample types, time points, analysis methods, within-farm compartments, and farms. In Farm A, two sampling events on different dates occurred in the circuit that houses 12–15 g perch. The first sampling event took place on June 25th, 2020 and consisted of collecting tank wall biofilm (samples 4–6), tank water from the same tank as the biofilm (samples 7–9), and biofilter water from the same circuit (samples 10–12). The sampling took place less than a week after the last tank cleaning. A second sampling took place on November 4th, 2020 and involved the collection of tank wall biofilm (samples 1–3), from a second tank within the same circuit, several weeks after the last cleaning of the tank. In farm B, sampling took place on November 23rd, 2020, that consisted of collecting tank wall biofilm from two tanks (samples 13–15 (tank 1) and 16–18 (tank 2)). Negative control samples were collected for the June 25th, 2020, sampling event but were not sequenced. The negative water control was filtered the same way as the on-site water samples, using distilled water instead of system water. The negative swab sample consisted of unpacking a swab on-site and placing it into a 2 ml tube without swabbing a surface. An overview of all samples is provided in Additional file 1.

### DNA extraction

Three DNA extraction methods were tested on pre-trial water and swab samples for optimal and consistent DNA yield and quality (Fig. 1C) because suboptimal lysis conditions can introduce stochastic bias against gram-positive bacteria, which have a thick, difficult-to-lysis outer wall. Tests included (1) the Purelink Microbiome DNA Purification Kit (Thermofisher), which is optimized for microorganism lysis, (2) the DNeasy PowerWater Kit (Qiagen), which is optimized for the isolation of genomic DNA from filtered water samples, and (3) phenol–chloroform extraction, which has been shown to produce high DNA yield from environmental samples [32]. The PowerWater kit produced inconsistent yields (results not shown), whereas the Phenol–Chloroform approach produced higher DNA yield but was contaminated by phenol carry-over, resulting in low DNA purity. The Purelink Microbiome kit consistently produced the highest quality and yield and was subsequently used for the study. Before extraction, frozen filters were crushed in a 2 ml Eppendorf tube with sterile 1000 ml pipette tips, increasing exposure to the lysis buffer. Bead-beating was performed in a TissueLyser set to full speed for 10 min per the manufacturer's instructions.

### Sequencing

#### Short amplicon

The performance of four amplicon-based 16S-targeting approaches was compared regarding amplification, read quality, and taxonomic and biological conclusions (Fig. 1D). Three amplicons designed for short-read Illumina sequencing included 16S variable regions V4 (primers 515F + 806R, hereafter referenced as "Earth"; [33]), V3-4 (primers 341F + 805R, hereafter referenced as "Miseq"; [34], and V1-3 (primers 27F [35] + 534R [36]; hereafter referenced as "27F_534R"; Table 1). One amplicon designed for long-read PacBio sequencing with the Shoreline StrainID kit included 16S, ITS, and 600 bp of the 23S gene [37]; Table 1). The Shoreline Complete StrainID kit uses a patented StrainID primer set.

**Table 1:** Primers used in this study

Targeted region	Short-hand	FW primer	FW primer sequence	FW primer length	FW melting temperatures	REV primer	REV primer sequence	REV primer length	REV melting temperatures	Amplicon length	Primer target species
16S: V3-4	MiSeq	341F	5′-CCT ACG GGN GGC WGC AG-3′	17 bp	58–60 °C	805R	5′-GAC TAC HVG GGT ATC TAA TCC-3′	21 bp	52–60 °C	~ 465 bp	Bacteria
16S: V4	Earth	515F	5′-GTG YCA GCM GCC GCG GTA A-3′	19 bp	64–68 °C	806R	5′-GGA CTA CNV GGG TWT CTA AT-3′	20 bp	54–58 °C	~ 300 bp	Bacteria & Archaea
16S: V1-3	–	27F	5′-AGA GTT TGA TCC TGG CTC AG-3′	20 bp	58 °C	534R	5′-ATT ACC GCG GCT GCT GG-3′	17 bp	56 °C	~ 500 bp	Bacteria
16S-ITS-23S	PacBio	–	Proprietory	–	–	–	Proprietory	–	–	2500 bp	Bacteria

Illumina MiSeq forward and reverse primers carried overhang adapters (5′-TCGTCGGCAGCGTCAGATGTGTATAAGAGACAG-Forward primer, 5′-GTCTCGTGGGCTCGGAGATGTGTATAAGAGACAG-Reverse primer) for compatibility with Illumina index and sequencing adapters

*FW* Forward primer, *REV* Reverse primer, *bp* Base pairs

Optimal amplification conditions suitable for all three short amplicons were determined by gradient PCR and reducing cycle number as much as possible. The PCR included 12.5 µl of KAPA HiFi HotStart Ready Mix (Roche, Switzerland), 5 µl of each primer (0.2 µM stock concentration), and 12.5 ng of DNA plus water to a total volume of 25 ul. PCR cycling numbers (14, 16, 18, 20, 22, and 25) were tested at annealing temperatures between 54 and 58 °C for all three primer pairs. Based on agarose gel electrophoresis evaluations of amplification success, the following protocol was derived: initial denaturation at 95 °C for 3 min, 20 cycles (denaturation at 95 °C for 30 s, annealing at 55 °C for 30 s, and extension at 72 °C for 20 s), and final elongation at 72 °C for 5 min. In addition to all samples, four positive controls (Zymobiomics microbial community standard (Zymo Research)) were amplified with this protocol. In addition, samples 19–21 were introduced at this step and are technical PCR-level replicates of sample 2 amplified with Earth primers. Notably, sample 19 yielded no sequencing data.

The preparation of 16S rRNA gene amplicons for the Illumina MiSeq System was designed and performed at the Next Generation Sequencing Platform, University of Bern, according to the "16S Metagenomic Sequencing Library Preparation" protocol (Illumina, art #15,044,223 Rev. B). The quantity and quality of the cleaned amplicons were assessed using a Thermo Fisher Scientific Qubit 4.0 fluorometer with the Qubit dsDNA HS Assay Kit (Thermo Fisher Scientific, Q32854) and an Agilent Fragment Analyzer (Agilent) with an HS NGS Fragment Kit (Agilent, DNF-474), respectively. Next, the index PCR step was performed as in the protocol except using IDT for Illumina DNA/RNA UD Indexes Set A (Illumina, 20,027,213), MyFi Mix (BIOLINE, BIO-25050) and the inclusion of a no template control (NTC). Then the amplicon libraries were assessed for quantity and quality, as described above, using fluorometry and capillary electrophoresis. The remainder of the protocol was followed, except that the library pool was spiked with 10% PhiX Control v3 (Illumina, FC-110-3001) to compensate for reduced sequence diversity. Finally, the library was sequenced at 2 × 300 bp using a MiSeq Reagent Kit v3, 600 cycles (Illumina, MS-102-3003) on the MiSeq sequencing instrument. The run was assessed using Illumina Sequencing Analysis Viewer 2.4.7. We used Illumina bcl2fastq conversion software v2.20 to demultiplex the library samples and convert generated base call files into FASTQ files. Short-read sequencing, before filtering, resulted in a total of 4,808,910 (27F_534R, samples only), 4,816,559 (Earth, samples only), and 5,149,263 (MiSeq, samples only) reads. Read numbers at all filtering steps are available in Additional file 1.

Raw data from Illumina amplicon sequencing were uploaded to the SRA NBI databank. Project ID and accession codes are documented in the “Availability of data and material” section.

#### Long amplicon

Long amplicon PacBio sequencing was performed at the Next Generation Sequencing Platform, University of Bern. The quantity and quality of the extracted DNA were assessed using a Thermo Fisher Scientific Qubit 4.0 fluorometer with the Qubit dsDNA HS Assay Kit (Thermo Fisher Scientific, Q32854) and an Agilent Femto Pulse system with an Ultra Sensitivity NGS kit (Agilent, FP‑1101), respectively. The DNA was then amplified using dual-unique barcoded primers targeting 16S-ITS-23S, using the StrainID kit from Shoreline Biome using strain ID Set Z, Barcodes T1-T16 (Shoreline Biome, STRAIN-Z-SLB). This approach involves a single-step PCR, consisting of primers containing the barcode and target-specific primer, generating amplicons ready for SMRTbell template prep and subsequent sequencing on the PacBio Sequel System. The protocol from input DNA to SMRT sequencing was followed according to the Shoreline Wave for PacBio Technical Manual, following all parameters for the Strain ID workflow. As well as the input DNA of interest, a no template control (NTC), and two community controls (ZymoBIOMICS Microbial Community DNA Standard and ZymoBIOMICS Microbial Community DNA Standard II (Log Distribution) (Zymo Research, D6305 and D6311, respectively) were included. The generated library was SMRT sequenced using a Sequel binding plate 3.0 and a sequel sequencing plate 3.0 with a 10 h movie time on a PacBio Sequel system on their own SMRT cell 1 M v3. The library was loaded at 9 pM and generated 15 Gb and 284,296 HiFi reads.

Raw data from PacBio amplicon sequencing were uploaded to the SRA NBI databank.

#### Shotgun metagenomics

Illumina shotgun metagenomics sequencing was performed at the Next Generation Sequencing Platform, University of Bern. The extracted DNA was assessed for quantity, purity, and length using a Thermo Fisher Scientific Qubit 4.0 fluorometer with the Qubit dsDNA HS Assay Kit (Thermo Fisher Scientific, Q32854), a DeNovix DS-11 FX spectrophotometer, and an Agilent FEMTO Pulse System with a Genomic DNA 165 kb Kit (Agilent, FP-1002-0275), respectively. Sequencing libraries were made using an Illumina DNA Prep Library Kit (Illumina, 20,018,705) in combination with IDT for Illumina DNA/RNA UD Indexes Set B, Tagmentation (Illumina, 20,027,214) according to the Illumina DNA Prep Reference Guide (Illumina, 10,000,000,254 16v09). Six PCR cycles were employed to amplify 30 ng of tagemented DNA. Pooled DNA libraries were sequenced paired-end on a NovaSeq 6000 SP Reagent Kit v1.5 (300 cycles; Illumina, 20,028,400) on an Illumina NovaSeq 6000 instrument. The run produced, on average, 159 million reads/sample. The quality of the sequencing run was assessed using Illumina Sequencing Analysis Viewer (Illumina version 2.4.7) and all base call files were demultiplexed and converted into FASTQ files using Illumina bcl2fastq conversion software v2.20.

Raw data from Illumina shotgun metagenomics sequencing were uploaded to the SRA NBI databank.

### Read processing

#### Short amplicon

Illumina short-reads were processed with the DADA2 v. 1.14.1 (Divisive Amplicon Denoising Algorithm 2) [38] pipeline. The DADA2 pipeline includes the inspection of read quality, quality filtering and trimming of reads, dereplication and error rate learning, sample inference for the determination of true sequence variants, merging of reads, construction of sequence table, removal of chimeric reads, and taxonomic assignment. Each primer dataset (Earth, MiSeq, 27F_534R) was first run independently through the DADA2 pipeline, then the Earth and MiSeq fastq files were combined into one file, which was processed with DADA2 (“Combined dataset”). Primers were removed with the DADA2 *trimLeft* function: *trimLeft* = *c*(19, 20) for Earth primers, *trimLeft* = *c*(17, 21) for MiSeq primers, and *trimLeft* = *c*(20, 17) for primer pair 27F_534R. Base pairs with a quality score below 30 at the end of the read were removed using the DADA2 *trimRight* function: *trimRight* = *c*(10, 90) for Earth and MiSeq primers and *trimRight* = *c*(30, 100) for primer pair 27F_534R, based on visual inspection of the quality plots (Additional file 1). All other *filterAndTrim* parameters were set at the default values. The DADA2 function *mergePairs* was applied in the individual and combined datasets to align the denoised forward reads with the reverse complement of the corresponding denoised reverse reads, producing a merged "contig" sequence. By DADA2 defaults, merged sequenced are only output if the forward and reverse reads overlap by at least 12 bp and are identical in the overlapped region. Unfortunately, for primer pair 27F_534R, after the removal of bp with a quality score less than 30 merging the noised forward reads and the reverse complement of the corresponding denoised reverse read was not possible as too many basepairs were removed. For the remove bimera denova step, the *minfoldParentOverAbundance* parameter was set to 5 for individual datasets and 8 for the combined dataset. The naïve Bayesian classifier method was used for all datasets, with the default *minboot* = 50 (bootstrap confidence values: Additional file 2).

After DADA2 filtering, the datasets retained the following amount of reads 3,195,326 (66.4% average) for the 27F_534R dataset, 4,044,627 (average 83.8%) for the Earth dataset, 4,212,750 (81.7%) for the MiSeq dataset, and 8,344,294 (81.5%) (per primer: Earth: 4,128,007 and MiSeq: 4,216,287) for the Combined dataset (Additional file 1). Reads from the technical samples (20 and 21) and mock communities were removed from the total amount of reads reported above.

Individual datasets were used to quantify individual primer pair read quality, while the Combined dataset was used to quantify alpha and beta diversity, technical replication reproducibility, MDS analysis, and enriched ASVs. Sequencing quality was analyzed using the percentage of reads with a Phred score equal to or larger than 30 for each sample type and primer. Microbial taxonomic alpha-diversity (intra-sample) was calculated using Richness and Shannon indices as implemented in the R package phylsoseq [39]. Species beta-diversity (inter-sample) was estimated using the Bray–Curtis dissimilarity metric, while the dissimilarity between groups was visually assessed with multidimensional scaling (MDS) plots.

#### Long amplicon

PacBio Shoreline long reads were demultiplexed without primer trimming, palindromes were removed, and reads with lengths smaller than 200 base pairs were filtered out using the SBAnalyzer software (Shoreline Biome).

#### Shotgun metagenomics

Illumina shotgun metagenomics reads were high quality, requiring no filtering.

### Taxonomic assignment

#### Short amplicon

Short-read data was assigned to taxonomic units with the SILVA v.138 gene reference database. After DADA2 processing, the Earth dataset contained 10,941 ASVs, with 196 ASVs assigned to the mock community sample and 3 ASVs assigned to both the mock community and samples. Of the 10,742 ASVs found within the samples, 10,501 were assigned to Bacteria, 14 to Archaea, 57 to Eukaryota, and 170 could not be assigned. For the MiSeq dataset, 6102 ASVs remained, with 20 ASVs assigned to the mock community and 2 ASVs assigned to both the mock community and samples. Of the 6080 ASVs found within samples, 6095 were assigned Bacteria, 2 to Archaea, 2 to Eukaryota, and 3 could not be assigned. For the combined dataset, 18,072 ASVs remained, with 236 ASVs assigned to the mock community and 3 ASVs assigned to both the mock community sample and samples. Of the 17,833 ASVs found within the sample, 17,822 were assigned to Bacteria, 16 to Archaea, 61 to Eukaryota, and 173 could not be assigned (Additional file 2).

Sample data were managed using the R package *phyloseq* (v1.30.0) (McMurdie and Holmes, 2013), and plots were generated using the R package *ggplot2* (v.2.2.1) [40].

#### Long amplicon

Long read data were taxonomically assigned with the Athena database v2.2, resulting in 99.3% of reads successfully classified (196,749 reads). An abundance table, a taxonomic classification list for each species, and a list of samples assigned to each read were created (Additional file 3). The initial goal was to compare output after running short- and long-reads through the DADA2 pipeline. However, the low read depths of the samples due to the mock community sample vastly outnumbering the samples during sequencing made this approach no longer possible. Therefore, the abundance table was analyzed manually for the spatial distribution of species.

#### Shotgun metagenomics

The raw reads of the metagenomics samples were classified according to their taxonomy using kraken2 [41]. This software classifies reads according to their best matching location in the taxonomic tree. Bracken was used to estimate the species abundance [42], using the taxonomy labels assigned by kraken2 to estimate the number of reads originating from each species present in the sample.

### Data analysis

#### Short amplicon

Read quality was assessed based on the percentage of reads with a Phred score greater than 30 for each primer.

Microbial taxonomic alpha-diversity (intra-sample) was evaluated with the Richness and Shannon indices implemented in the *microbiome* R package [43]. Species beta-diversity (inter-sample) was estimated with Bray–Curtis distances, using the ordinate function in the *phyloseq* package, to understand similarities and differences in community composition independent of primer choice, within-farm compartments, farm identity, and time point in the production cycle. The dissimilarity between samples was assessed by multidimensional scaling (MDS).

Community composition was analyzed between primers, replicates, sample types, and farms by comparing the relative abundance of the top 9 phyla, all other phyla (Other), and not assigned (NA).

ASV enrichments were analyzed with a PERMANOVA non-parametric multivariate test using the adonis function in the R package *vegan* (v.2.5.7) [44] to determine which ASVs were significantly enriched between tank samples of farm A and between farms. The top 20 enriched ASVs coefficients were plotted.

All analyses were completed in RStudio 1.4.1717 [45].

#### Long amplicon

The ten most abundant species were identified for each sample type per farm based on the total number of reads after both replicate reads were summed together. Abundance was compiled and plotted for these species to understand spatial and abundance distribution across sample types and farms. Markedly, some replicates have less than ten dots because the top species was only detected in one replicate.

#### Shotgun metagenomics

Phyla with at least 0.5% or more of the total reads were retained to analyze the overall community composition. A Sankey plot using the R *network3D* v.0.4 package [46] was plotted to compare the community composition across the domains. In addition, relative abundance bar graphs were plotted to quantify community composition variance at the replicate, sample type, and within-farm compartments.

All figures were prepared for publication using Adobe Illustrator 2021.

## Results

We used a tiered sequencing approach to analyze RAS microbial communities. Therefore, the results obtained from each sequencing dataset cannot be compared directly but complimentarily. Combining the datasets offers a more profound knowledge of the RAS than any one sequence approach could accomplish.

### Short amplicon

#### Read quality

**Fig. 2:**
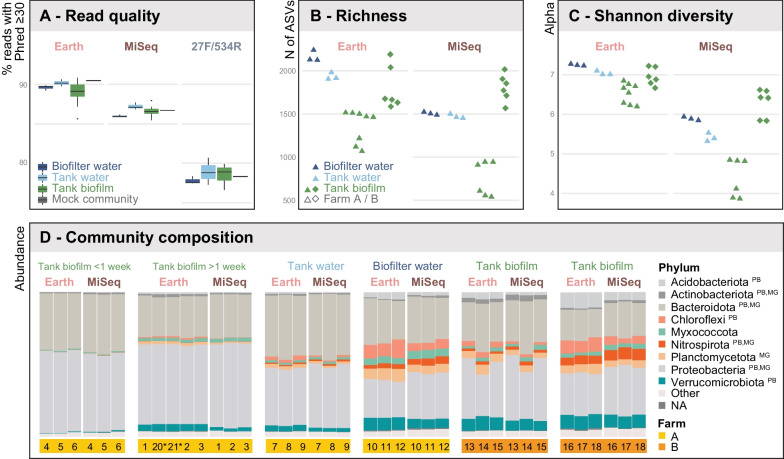
16S primer choice affects sequencing quality and diversity measures but not community-level results. **A** Read quality. Earth and MiSeq primers yielded reads with high sequencing quality, whereas primer set 27F/534R yielded lower read quality and was excluded from downstream taxonomic analyses. Between sample types, read quality was comparable for each primer set. **B** Richness. Earth primers yielded higher alpha richness based on ASVs across all sample types. In farm A, biofilter water featured the most ASVs, followed by tank water and, finally, tank biofilm. Richness in farm B's biofilm was as high (Earth) or higher (MiSeq) than farm A's biofilter water. **C** Diversity. Samples amplified with Earth primers displayed higher alpha diversity (Shannon index) than samples amplified with MiSeq primers. Patterns are overall similar to richness (panel B). The similar diversity patterns suggest that it would be possible to compare community studies using different primers at the relative scale. **D** Community composition. The biological and technical replicates (samples 2, 20–21) were highly similar in composition, suggesting that primer selection does not impact spatio-temporal findings at the phyla level and indicates that reproducible results can be obtained with short amplicon sequencing. Indications for succession can be seen in the tank biofilm samples, with increasing complexity from young to older biofilm. Finally, Farm B's tank biofilm samples resembled Farm A's biofilter water samples, suggesting that microbial communities with RAS become similar in complexity over time, potentially reaching a stable, mature state. Abbreviations after the phylum name indicate that the phylum was detected in other platform datasets; PB = PacBio, MG = Metagenomics

The overall read quality was satisfactory, with Earth, MiSeq, and 27F_534R primers producing Phred scores ≥ 30 for 89.3%, 86.6%, and 78.5% of reads, respectively (Fig. 2A). However, the lower read quality and the longer amplicon length of primer pair 27F_534R led to difficulties merging the forward and reverse reads using the merge function. Therefore, we decided to remove this primer from downstream analyses as it could not be processed in the same fashion as the other two primers.

#### Taxonomic assignment

Regarding taxonomic assignment, Earth and MiSeq amplicons performed similarly at a higher-level classification (e.g., phylum, order) but diverged at a lower-level classification (e.g., ASV). The Earth dataset identified 37 phyla, whereas the MiSeq dataset identified 34 phyla. However, the MiSeq dataset assigned 99 more genera at the genus level than the Earth dataset (470 vs. 371, respectively) (Additional file 2). Although the MiSeq primers could identify more taxa, Earth primers resulted in higher alpha diversity, both for richness (Earth: ranged: 1070–2240 compared to MiSeq: ranged 441–1962) and Shannon diversity (Earth: ranged: 6.12–7.32 compared to MiSeq: ranged 3.83–6.18) (Fig. 2C; Additional file 3). Within farm A, alpha richness was highest in biofilter water (Earth average: 2166 and MiSeq average: 1504), followed by tank water (Earth average: 1934 and MiSeq average: 1477) and tank biofilm, which was influenced by the age of the biofilm (Earth average: young 1135 vs. mature 1497 and MiSeq average: young 465 vs. mature 829). Within farm B, the tank biofilm average richness was similar between the two tanks (Earth: tank1 1793 vs. tank2 1805, MiSeq: tank1 1665 vs. tank2 1815). The Shannon diversity between sample types within farm A mirrored the pattern of richness, with biofilter water having the highest average diversity (Earth: 7.28, MiSeq: 5.88), followed by tank water (Earth: 7.07, MiSeq: 5.42), and the different aged biofilm samples (Earth: young 6.21 vs. mature 6.72, MiSeq: young 3.98 vs. mature 4.83). Farm B's tank biofilm samples had similar average Shannon diversity values (Earth: tank1 6.91 vs. tank2 7.10, MiSeq: tank1 5.59 vs. tank2 6.08) (Additional file 3).

#### Community patterns

**Fig. 3:**
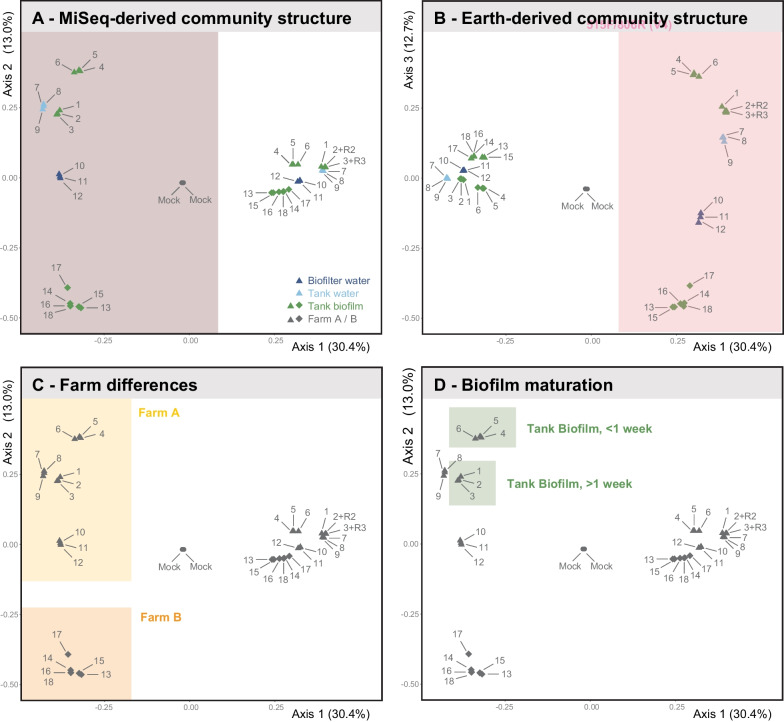
Impact of primers, sample types, farms, and production cycles on community patterns derived from 16S sequencing. Multidimensional scaling analysis using the Bray–Curtis distance matrix is visualized with MDS plots at the phylum level. **A** MiSeq primers. Axes 1 and 2 achieve a clear separation of all samples. Replicates cluster together closely to the point of overlapping, whereas sample types, compartments, and farms form separate clusters. **B** Earth primers. Earth primers achieve an identical overall pattern but in a distinct area of the morphospace. The mock communities cluster in the exact location independently of primer choice, further stressing the equivalence of primers at this level of analysis. **C** Farms. Farms separate along axis 2. Panel C visualizes this for MiSeq primers, but the pattern holds with Earth primers (Panel B). **D** Tank and Time. Both primers can distinguish the biofilm samples collected in farm A from two tanks of different operational times but within the same circuit, emphasizing that a short-read approach is sufficient to achieve fine-scale resolution at the community level

Amplicon choice did not affect the composition of the microbial community at higher taxonomic levels. Community composition for distinct sample types, replicates, and the derived spatio-temporal patterns were very similar between the two amplicons (Figs. 2D and 3A, B). Subtle biases for/against specific phyla (e.g., *Chloroflexi*, favored by Earth; *Myxococcota* and *Plantomycetota,* favored by MiSeq; Fig. 2D) did not affect the inferred overall community structure, which was virtually identical for both amplicons according to MDS analyses (Fig. 3A and B).

As revealed by the MDS analysis, multiple factors influence the community patterns, with environmental farm conditions being the primary driver (Fig. 3C), followed by sample type (Fig. 3A and B). Sample types featured distinct community compositions, but the same sample types did not necessarily cluster together (e.g., tank and biofilter water vs. biofilm). For example, farm B's tank biofilm was more similar to farm A's biofilter water than farm A's tank biofilm samples. Biofilm age also drove differences between community richness and dominating genera (Figs. 2D and 3D), with the young vs. mature biofilm consisting of 108 vs. 152 genera (Earth) or 126 vs. 190 genera (MiSeq), respectively. Upon further inspection, farm B's tank biofilm included 288 and 356 genera, whereas farm A's tank biofilm included 166 and 462 genera for Earth and MiSeq, respectively (Additional file 4).

#### Enriched ASVs

**Fig. 4:**
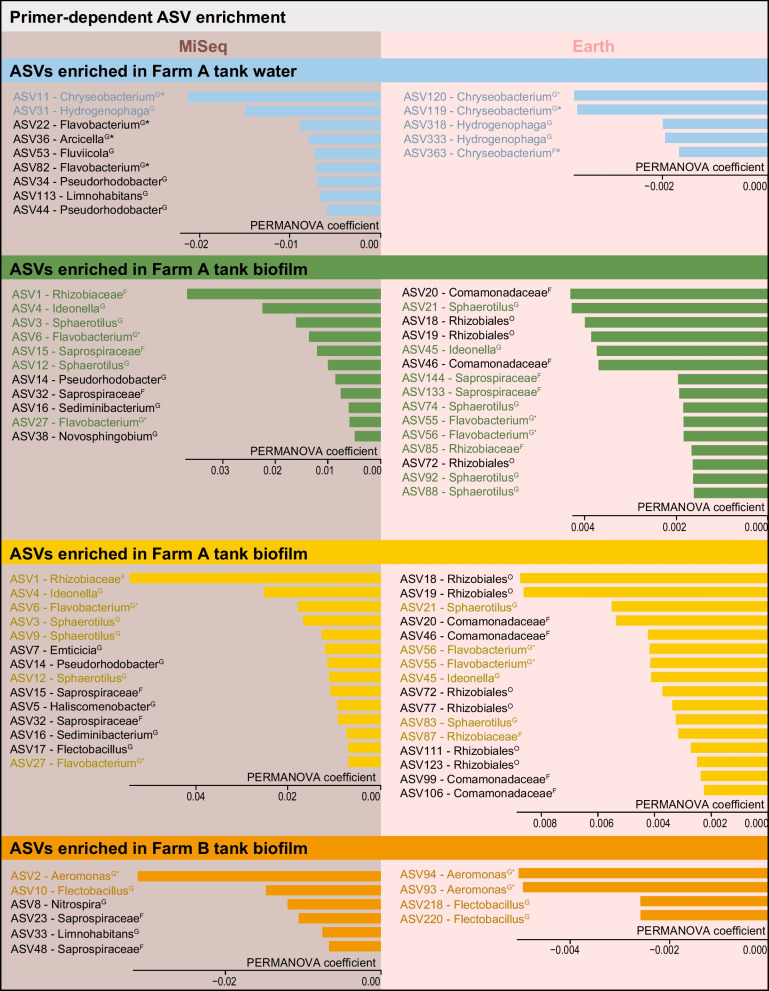
Taxonomic units unique for specific primers and/or compartmens according to 16S sequencing. Permanova coefficients indicate which ASVs are most characteristic for (but not necessarily most abundant in) a particular compartment. Uppercase captions indicate the lowest classified order (O = Order, F = Family, G = Genus). Taxonomic units containing aquaculture pathogens are marked with an asterisk. Primer pair differences (different emerge at the ASV level. We found that water and biofilm samples from the same farm and circuit differ in differentially enriched ASVs, which is vital for understanding taxa diversity and functional services within different sample types. Notably, both primers could identify pathogenic groups within the farms, e.g., Chryseobacterium, Flavobacterium, and Aeromonas

The differential enrichment of specific ASVs further drove the differences between communities and amplicons. Both primers agreed on differential enrichment of  *Chryseobacterium* and *Hydrogenophaga* in Farm A tank water, but they disagreed regarding the biofilm samples, with differential enrichment of *Rhizobiaceae* and *Ideonella* (MiSeq) vs. *Comamonadaceae* and *Sphaerotilus (Earth)* (Fig. 4). Considering the close clustering of these samples in morphospace (Fig. 3) these results could explain the taxa driving this separation. When comparing biofilm from farm A and farm B, ASVs differentially enriched in farm A were affiliated with *Rhizobiaceae* and *Ideonella* (MiSeq) and *Rhizobiales* and *Sphaerotilus* (Earth), while ASVs affiliated with members of *Aeromonas* and *Flectobacillus* (MiSeq and Earth) were differentially enriched in farm B (Fig. 4). Notably, Earth and MiSeq datasets agreed about the presence of taxonomic groups harboring pathogens, e.g., *Chryseobacterium*, *Flavobacterium*, and *Aeromonas*. These results show that at the level of ASVs, biases are introduced by primer choice.

### Long amplicon

The low number of reads obtained from the long-read amplicon approach (a consequence of harsh lysis conditions and over-sequencing of the mock community standard) prohibited overall community statistics approaches. Nevertheless, taxonomic conclusions of biological interest could be derived from the 10,041 reads obtained, which resulted in the identification of 204 species (Additional file 5).

**Fig. 5:**
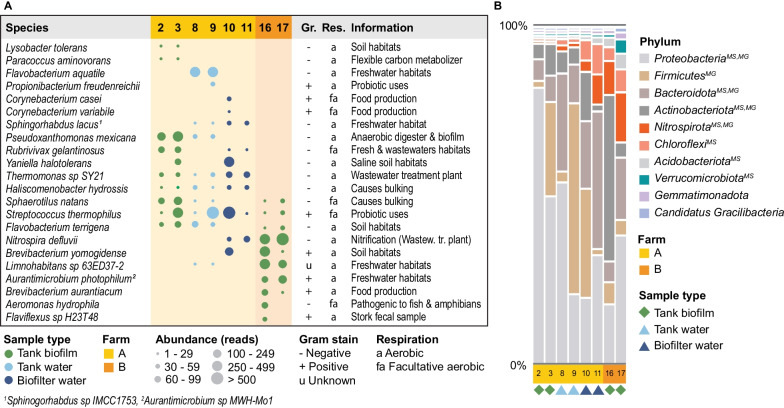
PacBio sequencing results (10 most abundant species identified in each compartment). The top ten species were identified by adding the reads from both replicates and then identifying the ten most abundant. The farms (yellow vs. orange) feature distinct communities, with distinct compartments within farm A sharing more species biofilm samples between farms. For example, *Haliscomenobacter hydrossis*, a species known to cause bulking, is ubiquitous and unique to farm A. Only three species, *Sphaerotilus natans*, *Streptococcus thermophilus*, and *Flavobacterium terrigena*, featured among the most abundant species in both farms. *Aeromonas hydrophila* was obtained from a tank in farm B that had an active Aeromonas spp. outbreak. The table further includes gram stain, respiration method, and additional information about the particular species. Updated species names are denoted as footnotes. Green diamonds represent tank biofilm, light blue triangles represent tank water, and dark blue triangles represent biofilter water. Uppercase captions indicate that the phylum was also detected in the other datasets; *MS* MiSeq, *MG* Metagenomics

Similar to the short-read data, species-level data obtained with long-reads emphasize the unique features of farms and, to a lesser extent, compartments (Fig. 5). Seventeen of the top enriched species were affiliated with biofilm samples. However, only five were shared between farms, including *Sphaerotilus natans*, a bacterium responsible for bulking, *Streptococcus thermophiles*, a commonly used probiotic bacterium, and *Nitrospira defluvii*, a bacterium that aids nitrification. Twelve species were exclusively detected in farm A, and four were specific to farm B. Within farm A, many of the species were detected in at least two compartments, except *Thermomonas sp.* SY21 and *Haliscomenobacter hydrosissis* that were detected in all compartments. However, *Lysobacter tolerans* and *Paracoccus aminovorans* were found explicitly in farm A's biofilm. The two water-type samples (biofilter and tank) from the same circuit featured similarities and differences when inspecting the top enriched species, with *Flavobacterium aquatile*, *Propionibacterium freudenreichii*, and *Limnohabitans sp*. 63ED37-2 detected in tank water, and *Corynebacterium casei*, C. *variable*, *Nitrospira defluvii*, and *Brevibacterium yomogidense* detected in biofilter water. Finally, in farm B's biofilm, *Aeromonas hydrophila*, a common secondary invader known to cause a broad spectrum of infections, was also differentially enriched.

### Shotgun metagenomics

**Fig. 6:**
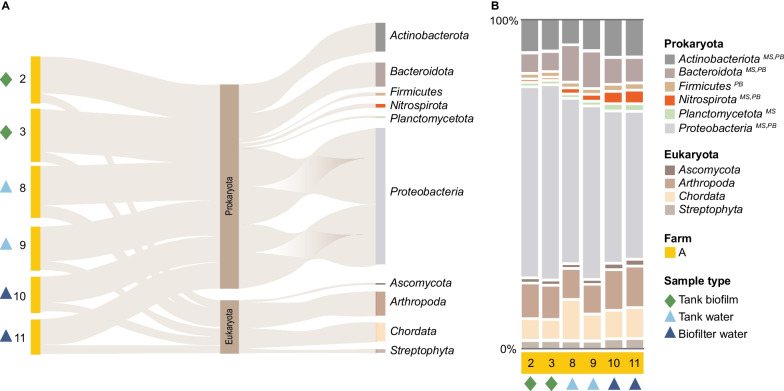
Shotgun metagenomics data for phyla with at least 0.5% or more of the total reads. **A** Sankey diagram displaying each sample's prokaryotic and eukaryotic phyla. The prokaryotic phyla are the dominant group in each sample, with Proteobacteria as the most abundant group. Eukaryotic phyla consist of (1) Arthropods likely introduced via the feed, (2) Chordata represented by the farm-raised European perch (Perca fluviatilis), (3) Streptophyta, which consists of green algae and land plants and (4) Ascomycota, sac fungi, representing the largest phylum of fungi. **B** Relative abundance plots illustrate the community composition similarities and differences between replicates, sample types, and within-farm compartments. Notably, Proteobacteria are less abundant in biofilter water than in tank biofilm. Uppercase captions indicate that the phylum was detected in the other datasets; *MS* MiSeq, *PB* PacBio. Green diamonds represent tank biofilm samples, light blue triangles represent tank water, and dark blue triangles represent biofilter water

The shotgun metagenomics data corroborated amplicon findings and extended the picture beyond prokaryotes (75.55%) and included eukaryotes (23.97%), archaea (0.24%), and viruses (0.24%) (Additional file 6). Focusing on phyla with at least 0.5% or more of the total reads, a dataset comprising 96.34% of all reads identified ten phyla. Three-fourths (75.26%) of these reads were assigned to prokaryotic phyla, indicating that competition with eukaryotic reads was not an issue (Fig. 6A). Shotgun sequencing agreed with the patterns detected by amplicon sequencing. The top phyla were *Proteobacteria* (54.72% of total reads), *Actinobacteria* (9.47% of total reads), and *Bacteroidetes* (8.05% of total reads) (Fig. 6A) for all samples (Fig. 6B). The eukaryote phyla comprised *Arthropoda* (10.29%), with fish food and spider colonies as the most likely source; Chordata (8.04%), with the farmed European perch (*Perca flavescens*) as the source; and *Ascomycota* (sac fungi); and *Streptophyta* (green algae and plants) (Additional file 6).

Among lower abundance phyla (0.50–0.08% of reads), 16 additional taxa, from a virus group to eukaryotic groups, were detected. The virus group was *Uroviricota,* a dsDNA-tailed bacteriophages virus. Four out of the six low-abundance bacteria phyla were also detected in other platform datasets. For instance, *Verrucomicrobiota, Acidobacteriota,* and *Chloroflexi* were detected in the MiSeq and PacBio datasets, and *Gemmatimonadota* was detected only in the PacBio dataset (Additional File 6). In addition, *Euryarchaeota*, a methane-producing archaean, was detected. The eukaryotes included invertebrates such as *Mollusca* (mollusks), *Echinodermata (starfish, sea cucumber and urchins, *etc*.)*, *Cnidaria (jellyfish, sea anemones, *etc*.)*, *Nematoda* (roundworms), and *Platyhelminthes* (flatworms). Additionally, *Basidiomycota* (fungus), *Chlorophyta* (green algae), and *Apicomplexa* (protozoan) were detected (Additional file 6).

As expected, pathogenic species were detected at even lower read abundance levels. The ten most abundant pathogenic bacteria included *Flavobacterium psychrophilum* (0.071%)*, Aeromonas veronii* (0.031%)*, A. hydrophila* (0.029%)*, F. branchiophilum* (0.026%)*, F. columnare* (0.015%)*, A. caviae* (0.014%)*, A. salmonicida* (0.005%)*, Vibrio vulnificus* (0.004%)*, V. parahaemolyticus* (0.004%), and *A. jandaei* (0.003%) (Additional file 6). The PacBio data for farm A's tank water samples also identified *A. hydrophila*, *A. salmonicida*, and *A. veronii*.

## Discussion

Microbial communities are the drivers and determinants of a successful RAS, but their composition, interactions, and spatio-temporal dynamics are often unknown. Targeted research in RAS is required to shed light on how these communities form, interact and provide services. On the one hand, such knowledge will lead to better management, innovative RAS design, and procedures to manipulate communities. On the other hand, such research will extend our understanding of the rules governing community ecology and evolution beyond controlled lab systems. In this paper, we compare the distinct layers and types of information obtained by distinct methodological approaches from short-read to shotgun metagenomics. We demonstrate that each method can present a cost-effective technique to monitor particular aspects of microbial communities within RAS.

### Primers, pipelines, and platforms

Variations in protocols concerning primers and amplification, sequencing platforms, quality filtering, and clustering parameters affect conclusions in microbial ecology. For example, primer bias will occur in any study that includes an amplification step. Understanding how these biases affect biological conclusions is essential, especially in a dynamic field such as aquaculture, where no consensus has been reached concerning methods. However, aquaculture microbiome research widely employs 16S rRNA sequencing as a cost-effective method for surveying microbial communities [1, 7, 10, 47, 48]. Primer selection for short-read sequencing is potentially the most influential step during aquaculture microbial community analysis, as primers directly select for or against specific groups based on the targeted 16S v-region [23, 27, 29, 49, 50].

In our study, primer pair 27F_534R underperformed, an unexpected result as this primer pair was successfully used with active sludge collected from a wastewater treatment plant [23]. We attribute this to our approach of co-sequencing all amplicons. Shorter fragments sequence more efficiently, and 27F_534R amplicons were likely out-competed by the shorter MiSeq and Earth amplicons [51]. This would explain the decrease in both read numbers and read quality with increasing amplicon size (Earth > MiSeq > 27F_534R; Fig. 2A). Therefore, the 27F_534R amplicon, which in theory would offer higher taxonomic resolution due to its increased length [9, 52], could still be adequate for future RAS samples, but should not be combined with shorter fragments during sequencing.

Minor differences in ASV richness between Earth and MiSeq primers did not impact the spatio-temporal patterns and biological conclusion, even though primer bias was detectable at higher taxonomic resolution (Fig. 4). This implies that community studies can potentially be compared at higher taxonomic levels even when different 16S rRNA primers were used. However, the significance of biases at high taxonomic resolution is somewhat uncertain, particularly since previous findings with the same primers differ at the family level. For example, Earth primers have been reported to underestimate the abundance of *Chloroflexi* and *Actinobacteria* in active sludge [23], while in our study, *Chloroflexi* appeared to be overrepresented with the Earth primers, while *Actinobacteria* was similarly represented by both primers (Fig. 2D).

In summary, our results suggest that short-read sequencing is adequate for exploring the spatio-temporal dynamics and community composition at higher taxonomic levels. Because of its low cost, ease of implementation and the availability of well-validated pipelines, 16S rRNA sequencing remains a powerful approach. It has the potential as a monitoring tool in larger-scale RAS farms that incorporate research and design projects into their annual budgets.

Long-read sequencing approaches are recommended to improve taxonomic resolution [53–56] and are desirable in a context where species-specific pathogen identification is relevant. A current drawback is that long-read methods require a large amount of high-quality starting material, thus making them unsuitable for environmental studies that often have low DNA yield [57] and high levels of amplification inhibitors. Also, including a mock community, as recommended for normalization [25], can compromise sequencing depth. The methodological requirements associated with environmental samples containing gram-positive bacteria, i.e., harsh lysis conditions, compromised our long-read approach that was further impaired when paired with high-quality community standards during sequencing. When aiming for high-quality long DNA fragments for long-read sequencing, lysis methods and the inclusion of mock standards require thorough optimization. We conclude that the taxonomic resolution of the PacBio approach is beneficial in exploring functional services and species identification, especially pathogenic ones. However, the approach might not be optimal for a large-scale spatio-temporal study that requires quantitative results and may suffer from challenges in DNA quantity or quality.

In contrast to the aforementioned short- and long-read approaches, amplification-free shotgun metagenomics are not impeded by primer bias. In addition, genome-wide information, read count and genome size can be used to calculate biogenomic mass—a proxy for biomass [58]. Species-independent functional profiling based on the presence or absence of genes is another benefit of metagenome data. Finally, shotgun metagenomics sequences all genetic information rather than just one taxon. RAS microbial ecosystems also harbor archaea [18], fungi [59, 60], and viruses [61], which all interact, compete for resources, and aid or deleteriously impact the system. Therefore, shotgun metagenomics represents the most thorough approach for characterizing RAS microbial communities.

In our study, most reads obtained by shotgun metagenomics were of microbial identity, but additional relevant taxa (especially viruses, archaea, and fungi) were detected (Additional file 6), confirming the effectiveness of the approach to provide a wholistic picture. Importantly, the metagenomics data mirrored the amplicon data, confirming the validity of the three sequencing approaches to reach relevant biological conclusions at higher taxonomic levels. The similarity in community patterns also supports our previous conclusion that the impact of primer bias in amplicon approaches is negligible at higher taxonomic levels of analysis. Shotgun approaches are, therefore, highly promising and could be further functionalized by stepping toward an RNA-focused metatranscriptomic approach [62, 63].

The selection of a suited bioinformatics pipeline for analyzing sequencing data is a critical step in microbial studies. Currently, six bioinformatics pipelines are commonly used for 16S rRNA gene amplicon data analysis [64], and all have the potential to introduce bias through sequencing errors [65]. DADA2 is an increasingly used pipeline that shows high sensitivity, can differentiate sequences at single-base resolution, and clusters sequences into ASVs [64]. ASVs are advantageous over OTUs because they represent true sample sequence variants, unlike OTUs that are derived from traditional clustering, which can be prone to sequencing errors and biases based on the algorithm used or the fixed identity threshold value. A large body of literature on aquaculture microbiomes works with operational taxonomic units (OTUs). However, aquaculture studies using ASVs are on the rise, including studies on host-microbiome interactions [66], microbial dynamics in RAS [10], and microbial dysbiosis during a *Tenacibaculosis* outbreak [67] that could provide relevant data for meta-analysis studies.

Our results support several conclusions on method choice with transfer potential to other studies. First, primer bias does not compromise higher-level spatio-temporal conclusions of 16S approaches as long as a sufficient number of high-quality reads are obtained. Importantly, relative differences in community composition between data obtained with different primers can safely be compared, whereas we recommend avoiding comparing absolute statistics of microbial communities analyzed with different primers or lower taxonomic levels. Second, the requirements and challenges of long-read approaches complicate quantitative spatio-temporal community analyses but have value in species-level identification. Lastly, our results agree with other studies on the benefits of hybrid sequencing approaches [68–71]. The combination of three different sequencing methods yielded an in-depth overview of spatio-temporal dynamics and species-level information that would otherwise have been difficult to obtain.

### Community composition

Combining three different sequencing approaches allows for an in-depth assessment of microbial communities, including potential functional aspects. The dominating phyla in both the short-read amplicon (Fig. 2D) and the shotgun approach (Fig. 6) were *Bacteroidetes* and *Proteobacteria,* which agrees with previous short-read RAS studies (marine RAS: [7, 10, 12, 72]; freshwater RAS: [47]). *Bacteroidetes* contain species that are specialized in the degradation of complex polymers and the cycling of carbon and protein-rich substance [73, 74] and tend to be attached to particles or surfaces [7]. For example, *Flavobacteria*, a class in *Bacteroidetes*, were recently discovered to play a major role in nitrous oxidation–reduction, the final step of denitrification [75]. *Proteobacteria* are a diverse phylum containing nitrifying and denitrifying genera [18], which play a major role in nutrient recycling and remineralization of organic matter [76–78], essential steps for the operation of RAS.

A key finding of this study is the strong impact of the sample site and sample type on results and conclusions, as seen across the different datasets. Differences between biofilm and water samples have been reported before, e.g., for a sole RAS [10], a flow-through lumpfish farm [8], and an Atlantic salmon RAS [6], albeit only at higher taxonomic resolution. We show that overall community composition and species presence/absence differ not only between biofilm and water, but also between different compartments of the same circuit and between biofilm successional stages. Within the MiSeq data, differentially enriched ASVs were detected between the tank water and biofilm. The tank water differentially enriched ASVs belong to the genera *Chryseobacterium*, *Flavobacterium*, and *Hydrogenophaga*. *Chryseobacterium* [79] and *Flavobacterium* [81, 82] include opportunistic pathogens that impact fish health, resulting in devastating losses in wild and farmed fish stock worldwide. Furthermore, *Chryseobacterium* species are suspected of playing a role in spoilage [82] and being multidrug-resistant [83], which is a danger to both animals and humans. Differentially enriched ASVs in tank biofilm were *Rhizobiales*, *Ideonella*, *Comamonada*, and *Sphaerotilus*, which are involved in nutrient recycling processes or water quality. Notably is *Ideonella*, a small genus group composed of four species, with one species, *Ideonella sakaiensis*, capable of degrading PET, a polymer widely used in food containers, bottles, and synthetic fibers [84]. Since plastics are used in RAS for biofilter media (e.g., biofilter carriers), the presence of a potentially plastic-degrading species has implications for replacement and repair costs. The PacBio data showed that certain species were compartment-specific. For example, *Lysobacter tolerans* only occurred in the tank biofilm samples. They are capable of producing peptides that can damage the cell walls or membranes of other microbes and are regarded as an untapped source for producing novel antibiotics [85]. Species only found in the tank water included *Flavobacterium aquatile,* a species typically found in waters containing a high percentage of calcium carbonate—a characteristic of many Swiss waterways [86]—and *Propionibacterium freudenreichii*, an essential bacteria in the production of Emmental cheese, a Swiss cheese [87]. This type of information is essential for managers when choosing the type of sample to take for monitoring and diagnostic purposes and at the same time is promising regarding the use of RAS as models for spatiotemporal community dynamics.

Another key result is the major impact of community maturation state on biofilm community results. The biofilm succession process entails a non-random process controlled by attachment events, movement, and cellular interactions that induce the non-random spatial organization of biofilms [88]. As biofilms develop, they increase in volume and surface area, creating gradients of conditions that open niches, e.g., for anaerobic species [89]. This additional habitat complexity increases species richness and functional services, such as degrading organic compounds, cycling of nutrients, or preventing the establishment of pathogenic species through niche exclusion. At the same time, biofilms may act as a pathogen haven and/or reservoir [90]. For example, *Aeromonas hydrophila* (found in farm B, Additional files 5 and 6) can form thick layers that allow them to evade disinfection or antibiotic treatments [82, 91] while enabling the spread of antimicrobial resistance genes [92]—an area we are excited to explore with future shotgun metagenomics data.

In aquaculture management, biofilms are regularly removed during cleaning procedures, leaving them in a continuous state of recolonization. The impact of the removal and the resulting successional processes on ecological functions and animal health in RAS is unknown, but frequent disruption may potentially open up niches to pathogenic species while preventing the establishment of beneficial slow colonizers. A study by Rampadarath et al*.* [93] showed that within the first 24 h of biofilm formation, *Proteobacteria* microbials were the most dominant, followed by *Firmicutes*, *Bacteroidetes*, *Chloroflexi*, *Actinobacteria*, and *Verrucomicrobia*. Some of the most prominent bacterial fish pathogens are distributed across the phyla *Proteobacteria* and *Bacteroidetes*, which are early colonizers. In our data, we find the beneficial *Nitrospira defluvii* only in mature samples (farm A: biofilter water and farm B: tank biofilm), suggesting that these species are late colonizers and that frequent biofilm removal could prevent their establishment and negatively impact denitrification.

We look forward to further disentangling the impact of frequent disruption and recolonization processes on biofilm communities and identifying factors promoting the establishment of healthy communities after a disruption. Identifying key steps towards colonization with beneficial communities could reduce start-up and operation costs [1], prevent the establishment of pathogens [94], and lead to healthier stock [95].

Finally, community patterns between farms are suggestive of an "island-biogeography" effect, where distinct communities develop in largely isolated habitats. Other aquaculture facilities studies have reported such effects [1, 96]. The long-read data clearly distinguishes farm communities (Fig. 5), with *Haliscomenobacter hydrossis* (i.e., causes bulking) [97] and *Streptococcus thermophiles* only being present in farm A. Furthermore, the between-farm biofilm communities only had three species in common: *Sphaerotilus natans*, another bulking species [98], *Streptococcus thermophilus*, and *Flavobacterium terrigena*. In the present case, the conclusion is that farm conditions such as design, management styles, source water, environmental parameters (e.g., temperature, salinity [99], pH) in addition to farmed species, fish feed, and nutrient concentrations [10, 103], combined with stochastic assembly processes of dispersal and colonization [100], supersede the continued exchange of microbial communities through the regular delivery of juveniles from farm A to farm B.

### Disease and health

Understanding the potential pathogenic risks within a RAS is vital from the perspective of economic success but also to preserve animal health and wellbeing. The emergence and spread of pathogens accompany the current growth and rapid progress of aquaculture. Aquaculture disease outbreaks can be catastrophic to the industry, causing an estimated worldwide loss of more than US$6.0 billion per annum [101].

The shotgun metagenomics approach detected various pathogenic species in farm A that pose a risk to fish health and can ultimately result in disease outbreaks (Additional files 5 and 6). *Flavobacterium psychrophilum* (0.09% of total reads), the causative agent for bacterial coldwater disease, and *Aeromonas veronii* (0.04% of total reads), causing freshwater fish sepsis and ulcer syndrome, were the most abundant pathogens detected. Interestingly, these species are not typically associated with perch but with freshwater salmonid fish, such as rainbow trout (*Oncorhynchus mykiss*). However, a potential risk in animal farming is the emergence of spillovers and strains with altered host specificities. In addition, ubiquitous pathogens known to infect a wide range of freshwater fish, including perch, were detected across both systems at lower abundances and predominantly in tank water, including *Flavobacterium branchiophilum,* the causative agent of bacterial gill disease; *Aeromonas hydrophila,* the causative agent of motile aeromonas septicaemia; and *Flavobacterium columnare,* the causative agent of columnaris disease.

The development of nonpharmaceutical controls for pathogens in animal farming is vital for animal and public health. Antibiotic resistance poses one of the greatest human health and sustainability challenges of the 21st century [102]. Antibiotics have fostered the emergence of resistance genes and the promotion of horizontal gene transfer and mutagenesis in aquatic bacteria [103]. One proposed alternative method is bacteriophage therapy, which uses naturally-occurring bacteriophages to target specific bacteria species or strains of bacteria, such as *Ackermannviridiae* sp. or *Myoviridae* sp. Both phage groups were present in the studied farms (Additional file 6). However, phage therapy is still in its infancy, with only a handful of successful phage therapies for the 150 different bacterial pathogens of farmed and wild fish (e.g., *A. hydrophila* in loaches, *F. columnare* in catfish, and *F. psychrophilum* in rainbow trout) [104]. Our results show the potential of shotgun genomics to support the development of additional innovative phage therapies or other pathway-based disruptive measures.

## Conclusion

Our results show that microbial communities in RAS are highly dynamic and site-specific despite the permanent circulation of water throughout the system. Additionally, management routines create a state of continuous succession and recolonization, especially for biofilm communities. Finally, commonly used 16S primers can detect spatio-temporal development and dynamics across RAS compartments, sample types, and farms, but cannot provide the resolution required for species or strain identification, which is critical knowledge for RAS managers.

The results presented here contribute to quantifying the microbial community and dynamic and complex interactions in RAS. Further research of microbial communities in aquaculture is necessary to harvest the full power of these micro- —but mighty—organisms during farm management (e.g., during biofilter start-up or disease prevention), to extract basic biological principles (e.g., the link between environmental stressors and microbiome dysbiosis), and to clarify medically relevant interactions (e.g., between host-microbiome-environment interaction and disease development).

## Supplementary Information

**Additional file 1.** Information regarding samples, primers used for amplification, and reads per samples.

**Additional file 2.** Information regarding ASVs for each dataset and the assigned taxa.

**Additional file 3.** Information regarding the alpha values for each dataset.

**Additional file 4.** Information regarding taxa identified during the different maturation stages of biofilm.

**Additional file 5.** Information regarding taxa identified with the PacBio sequencing data.

**Additional file 6.** Information regarding taxa identified with the Illumina shotgun metagenomics data.

## Data Availability

The datasets supporting the conclusions of this article are available in the SRA NBI databank: Illumina short-amplicon: project ID: PRJNA757614; accession codes between SRR18029926–SRR18029932 and SRR18029942–SRR18029955). PacBio long-amplicon: project ID: PRJNA757614; accession codes between SRR18029933-SRR18029941; and Illumina Metagenomics: project ID: PRJNA757614; accession codes between SRR20005812–SRR20005817.

## Code availability

Code used to analyze data is available at 10.5281/zenodo.7553703. The abundance table output of the Shoreline PacBio sequencing is included within the article and its supplemental files.
